# Gender disparity in prestigious speaking roles: A study of 10 years of international conference programming in the field of gambling studies

**DOI:** 10.1371/journal.pone.0286803

**Published:** 2023-06-22

**Authors:** Eva Monson, Kimberly Ng, Hannah Sibbick, Djamal Berbiche, Adèle Morvannou

**Affiliations:** 1 Faculty of Medicine and Health Sciences, Université de Sherbrooke, Longueuil, Québec, Canada; 2 Independent Researcher, Montreal, Quebec, Canada; 3 Faculté de Médecine et des Sciences de la Santé, Département des Sciences de la Santé Communautaire, Université de Sherbrooke, Sherbrooke, Canada; Virginia Tech Carilion School of Medicine, TAIWAN

## Abstract

The objective of this study was to examine the distribution of prestigious speaking roles by gender at gambling studies conferences to better understand the state of gender representation within the field. Keyword searches were conducted in the fall of 2019. A total of 16 conferences that occurred between 2010–2019 and comprising 882 prestigious speaking opportunities were included. Quantitative analysis (i.e., t-tests, chi-squared posthoc tests) was undertaken to evaluate the representation of women speakers and if proportions were the same across genders for speakers. There were significantly less women than men within prestigious speaking roles at gambling studies conferences with only 30.2% of speakers being women (*p* < .001). This underrepresentation of women was consistent across conference location, speaker continent, speaker role, time, and across the majority of conferences. Women held prestigious speaking roles less frequently than men (*M* = 1.48 vs. 1.76; *p* < .001). A 9 to 1 (*p* < .001) ratio of men to women was found among top 10 most frequent prestigious speakers. While there was a higher proportion of women than men among student speakers and there was no significant gender disparity among early career researchers, there was a significantly lower proportion of women than men among speakers who hold more senior academic positions. There is an issue of gender disparity in prestigious speaking roles at conferences within the gambling studies field. This study highlights the need to counteract gender disparities and make room for diversity within the field.

## Introduction

Presenting at academic conferences, especially in prestigious speaking roles such as giving keynote, invited, plenary, and opening/closing addresses, is an important avenue for academics to expand research networks, gain visibility, and distribute their research outputs [1–3]. These highly coveted opportunities to present also play an important role in academic advancement, particularly for early career researchers and those seeking tenure (i.e., permanent, stable employment) [3,4].

Previous literature has demonstrated that women are underrepresented as speakers at academic meetings and conferences across a wide range of fields [5–9]. Recent studies have found that women comprise less than 30% of speakers at conferences and symposia in other fields [9–11], including one study that reported that more than a third of panels at medical conferences consisted only of men [5]. Women are also less likely to submit abstracts for oral presentations over poster presentations and less likely to be granted an oral presentation when they did apply [3,12].

The underrepresentation of women is also an issue within academia more broadly. Women are cited less often [13,14], receive fewer awards and prizes [15], publish fewer articles, and are less likely to receive top tier research funding [1]. Women hold fewer positions on editorial boards and are less likely than men to be requested as peer reviewers [2,16–18]. Beyond limiting women’s opportunities for integral components of career development like visibility, recognition, and mentoring, the reduced visibility of high-quality research by women also hinders scientific advancement and proliferates scientific discourse that lacks important diversity [1,2,9,11].

The gambling studies field has been described as multidisciplinary [19], though it remains dominated by certain disciplines, specifically psychiatry, neuroscience, psychology, and health sciences [20,21]. The field has also been criticized for being insular, uncritical, and homogeneous [22,23]. Specific to issues of gender disparity, research in the gambling studies field has demonstrated that the ratio of men to women among the top ten most cited researchers is 8 to 2 [20]. Despite some interest in gender disparity within the field [20,24], to our knowledge no research has examined the gender representation in prestigious speaking roles at conferences in the gambling studies field. The objective of this study was to examine the distribution of prestigious speaking roles by gender at gambling studies conferences to better understand the state of gender representation within the field.

## Methods

### Data collection

Google searches were conducted in English using a combination of the following keywords: “gambling,” “conference,” “congress,” and “international” were conducted in the fall of 2019. Each conference was reviewed for conformity to the following inclusion criteria: (1) having occurred in the past 10 years (2010–2019); (2) having an academic/research presence; (3) having international participation, and (4) having a minimum of two iterations. Eighteen conferences were initially identified, from which two were excluded; one due to its lack of academic speakers and another because it had only one iteration. For the remaining conferences, a search was undertaken to obtain official programs (i.e., brochures, schedules) for each conference iteration. When programs were not available on existing conference websites, secondary online sources were used (e.g., wayback machine/archive.org) and, if necessary, current conference organizers were contacted via email.

### Data extraction

The following information was extracted from each conference program: conference location (country; recategorized into continent) and year, as well as speakers’ names, affiliations, pronouns, countries (recategorized into continent), roles (i.e., keynote address, plenary session speaker, invited presenter, opening/closing address), and career stage (i.e., student, early career [up to 10 years after receiving PhD], non-early career). Speakers were only recorded once per conference (i.e., if a speaker presented multiple times at the same conference, only one presentation was considered in the analysis). Data was extracted by one author (KN) in consultation with the two lead authors (EM and AM).

#### Gender

Pronouns were used as a proxy for gender. When information was not present within the conference program (e.g., within speaker bios/blurbs), further online searches were conducted by examining publicly available biographies via university/personal websites, published articles, resumes, LinkedIn, ResearchGate, ProQuest (i.e., PhD dissertation search), and news articles.

While this study did not limit its data extraction from conference programs to binary pronouns, all conference speakers used either he/him or she/her pronouns in available materials. We refer to those using she/her pronouns as women and he/him pronouns as men and recognize that this generalization might not be accurate in all cases.

### Analysis

T-tests and chi-squared (χ2) tests were conducted to test if proportions were the same across genders for speakers. Posthoc tests were run to specifically understand on what variables there are differences according to gender. Data were analyzed using SAS [25].

## Results

The final sample included 16 international gambling studies conferences (Table 1) comprising a total of 105 conference iterations, for which 101 programs were located. A total of 882 prestigious speaking opportunities by 529 distinct speakers were analysed for this study (Table 2). A total of 179 women and 350 men were identified. We did not find any speakers using pronouns other than she/her or he/him. Missing data were, for the most part, minimal (i.e., <2%) for all variables, except for career stage which was 19.2%.

**Table 1:** Gender representation by conference.

Conference Name	Years Included	Total Number of Speakers	Women (%) *N* = 266	Men (%) *N* = 616	*p* Value
Alberta Gaming Research Institute’s Annual Conference*	2019, 2018, 2017, 2016, 2015, 2014, 2013, 2012, 2011, 2010	203	69 (34.0)	134 (66.0)	<.001
Annual National Center for Responsible Gambling^1^ Conference on Gambling and Addiction*	2019, 2018, 2017, 2016, 2015, 2014, 2013, 2012	101	30 (29.7)	71 (70.3)	<.001
Annual Nevada State Conference on Problem Gambling*	2019, 2018, 2016, 2014, 2013, 2012, 2011, 2010	20	7 (35.0)	13 (65.0)	.18
Asia Pacific Conference on Gambling & Commercial Gaming Research*	2013, 2012, 2011	15	3 (20.0)	12 (80.0)	.02
Asian Pacific Problem Gambling and Addictions Conference^2^	2015, 2011	8	1 (12.5)	7 (87.5)	-
East Coast Gaming Congress	2019, 2017, 2016, 2015, 2014, 2012, 2011, 2010	56	3 (5.4)	53 (94.6)	<.001
European Conference on Gambling Studies and Policy Issues	2018, 2016, 2014, 2012, 2010	62	12 (19.4)	50 (80.7)	<.001
GambleAware Conference	2019, 2018, 2017, 2016, 2015, 2014, 2013	40	26 (65.0)	14 (35.0)	.058
Gambling Harm Conference	2018, 2016, 2014	20	6 (30.0)	14 (70.0)	.074
International Conference on Gambling and Risk Taking*	2019, 2016, 2013	20	2 (10.0)	18 (90.0)	<.001
International Gambling Conference	2018, 2016, 2014, 2012, 2010	42	11 (26.2)	31 (73.8)	.002
International Multidisciplinary Symposium	2018, 2014	36	11 (30.6)	25 (69.4)	.02
National Association for Gambling Studies Annual Conference	2019, 2018, 2017, 2016, 2015, 2014, 2013, 2012, 2011, 2010	88	30 (34.1)	58 (65.9)	.003
National Conference on Gambling Addiction & Responsible Gambling*	2019, 2018, 2017, 2016, 2015, 2014, 2013, 2012, 2011, 2010	49	15 (30.6)	34 (69.4)	.007
New Horizons in Responsible Gambling Conference	2019, 2018, 2017, 2016, 2015, 2014, 2013	11	2 (18.2)	9 (81.8)	.035
Responsible Gambling Council Discovery Conference	2019, 2018, 2017, 2016, 2015, 2014, 2013, 2012, 2011, 2010	111	38 (34.2)	73 (65.8)	<.001

^1^Name was changed to International Center for Responsible Gambling in on January 1^st^, 2020.

^2^Asian Pacific Problem Gambling and Addictions Conference did not have a large enough sample of women to perform statistical analyses.

*Conferences were contacted to inquire about missing programs. The program for 2011 was missing for the Annual NCRG Conference on Gambling and Addiction. The programs for 2017 and 2015 for the Annual Nevada State Conference on Problem Gambling were missing. The program for the 2010 Asia Pacific Conference on Gambling & Commercial Gaming Research was missing.

**Table 2:** Speaker demographics.

Speaker Information	Demographic Variables	Frequency	%
Gender	Women	266	30.2
	Men	616	69.8
	Non-Binary	0	0
Speaker Role	Keynote	197	22.3
	Plenary	256	29.0
	Invited Other	246	27.8
	Opening/Closing	179	20.3
Career Stage	Student	30	3.4
	Early Career	142	16.1
	Non-Early Career	542	61.3

### Gender representation

**Fig 1:**
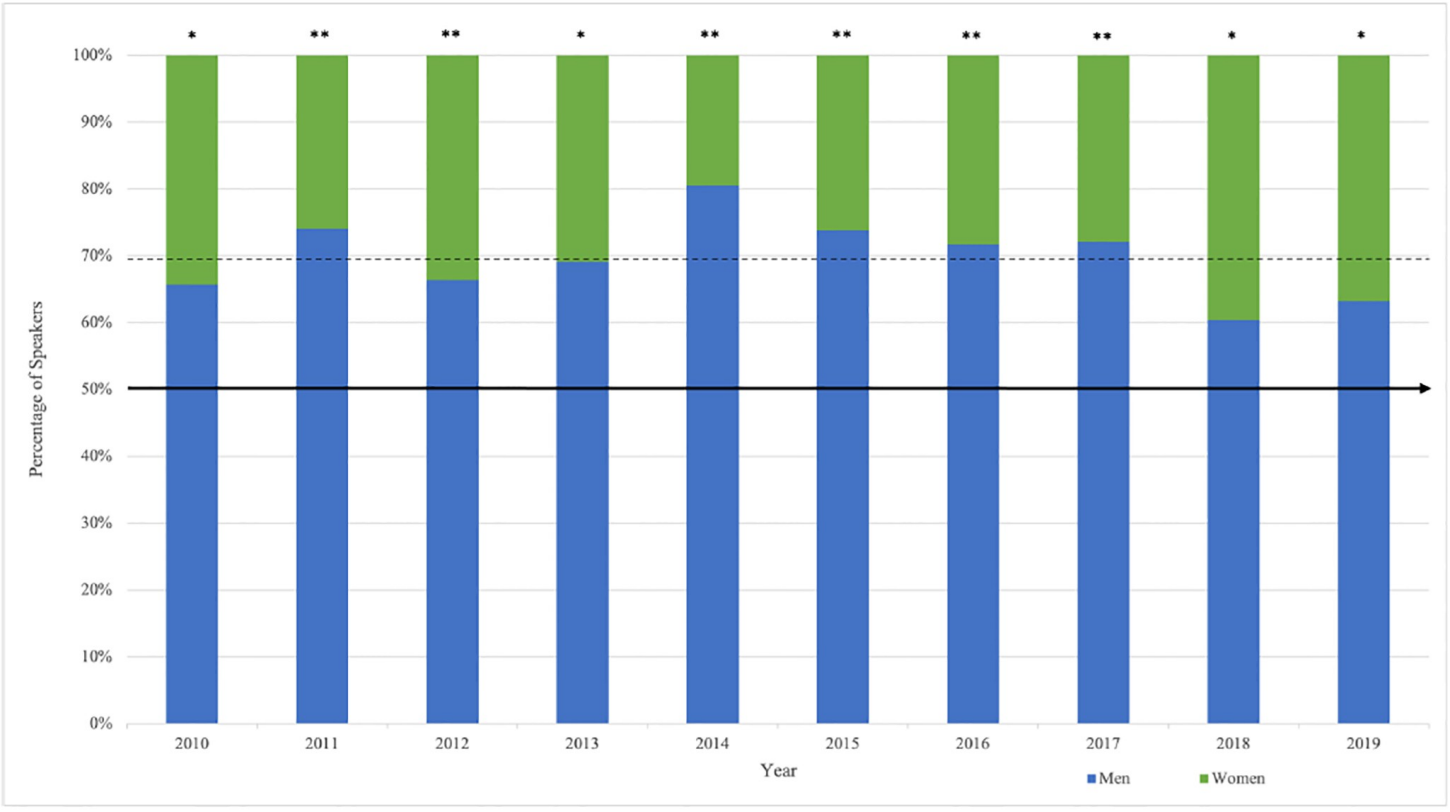
Gender representation by year. Note: * = *p* < .05, ** *p* < .001.

Overall, significantly less prestigious speaking roles at international gambling studies conferences were occupied by women compared to men: only 30.2% of speakers were women $X^{2}$ (1, *N* = 882) = 138.89, *p* < .001. Significant differences in gender representation remained stable over the 10 years of the study period (Fig 1). There were also significantly less women than men for each type of speaking role: keynote (22.3% women), $X^{2}$ (1, *N* = 197) = 49.75, *p* < .001; plenary (29.0% women), $X^{2}$ (1, *N* = 254) = 33.32, *p* < .001; invited other (27.8% women), $X^{2}$ (1, *N* = 246) = 13.67, *p* < .001; and opening/closing address (20.3% women), $X^{2}$ (1, *N =* 179) = 52.56, *p* < .001.

### Gender representation by location and conference

**Fig 2:**
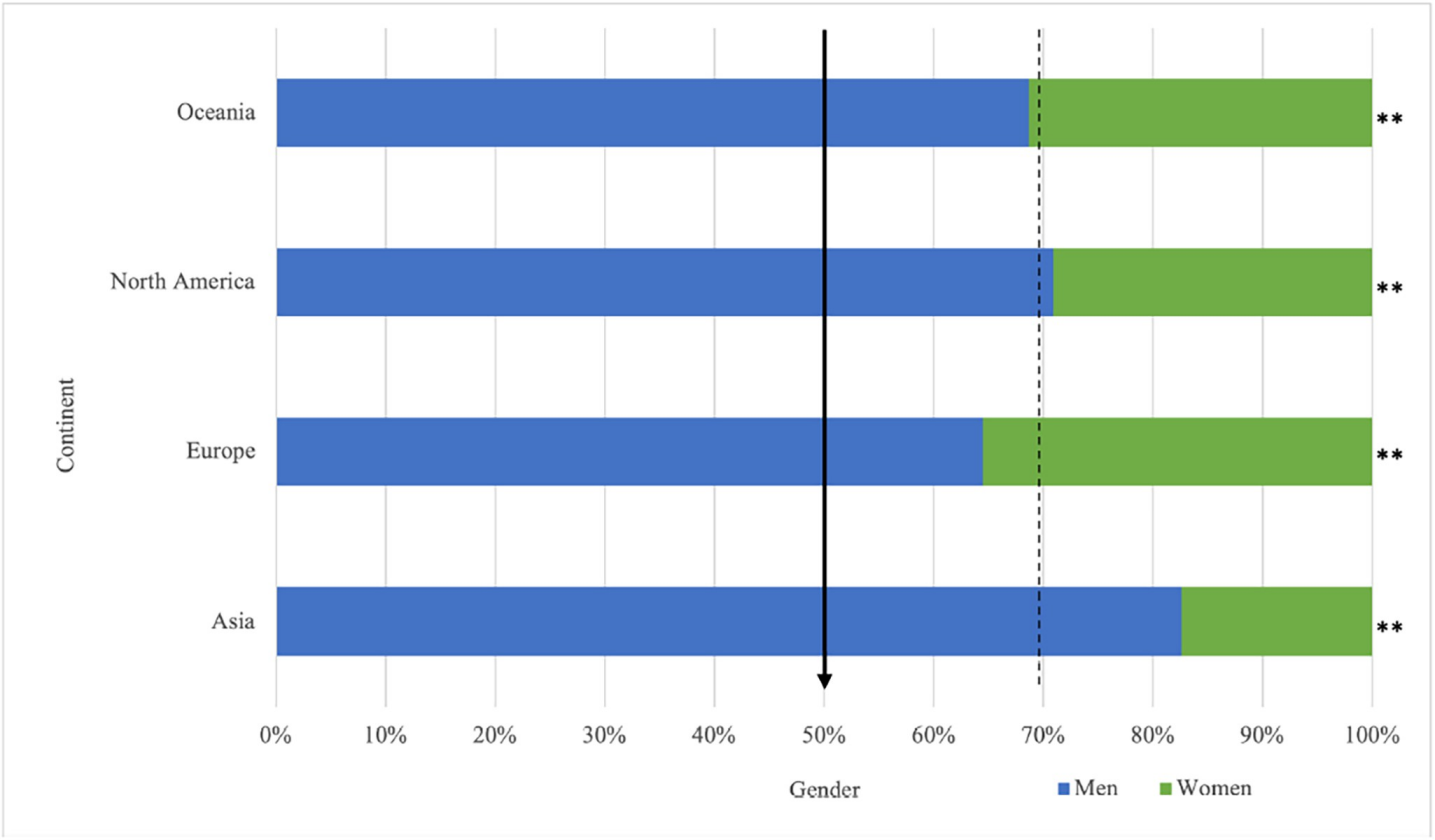
Gender representation by conference continent. Note: * = *p* < .05, ** *p* < .001.

**Fig 3:**
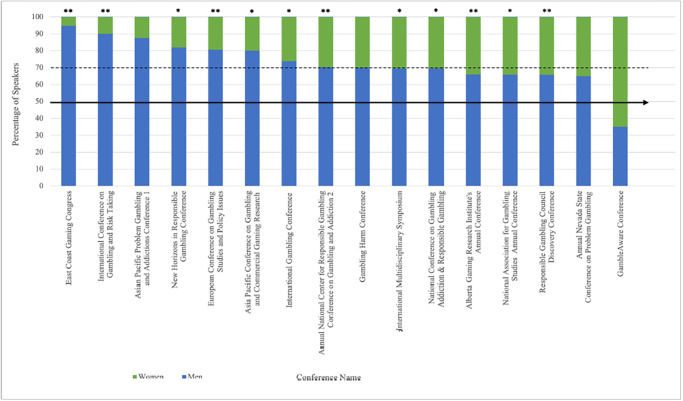
Gender representation by conference. Note. * = *p* < .05, ** *p* < .001. ^1^ The Asian Pacific Problem Gambling and Addictions Conference did not have a large enough sample of women to perform statistical analyses. ^2^ Name was changed to International Center for Responsible Gambling in on January 1, 2020.

There were significantly less women than men across all conference continents (Fig 2). On average, European conferences had the highest proportion of women (35.5%, *p* < .001), followed by those taking place in Oceania (31.3%, *p* < .001) and North America (29.1%, *p* < .001), whereas those taking place in Asia had the lowest proportion of women (17.4%, *p* = .002). For speaker continent, there were significantly less women than men across all continents where analyses could be conducted (i.e., Asia, Europe, North America, Oceania; Table 3). Despite the samples for Africa and South America being too small to analyse, a pattern remained with 100% of presenters being men. Women, on average, held prestigious speaking roles less frequently than men (*M* = 1.48 vs. 1.76; *p* < .001). There is an even larger divide when analysing the top ten most frequent speakers wherein the ratio of men to women is 9 to 1 (*p* < .001). Women were significantly less likely than men to occupy prestigious speaking opportunities for 12 of the 16 included conferences, no differences were found for three conferences, and one conference did not have a large enough sample of women to perform statistical analysis (Fig 3).

**Table 3:** Gender representation by speaker continent.

Continent	Total Number of Speakers	Women (%)	Men (%)	*p* Value
Africa	3	0	100	-
Asia	15	20.0	80.0	.02
Europe	153	33.9	66.1	<.001
North America	531	27.3	72.7	<.001
Oceania	165	40.0	60.0	.01
South America	3	0	100	-

Analysis could not be run for Africa or South America due to small sample size.

### Gender representation by career stage

**Fig 4:**
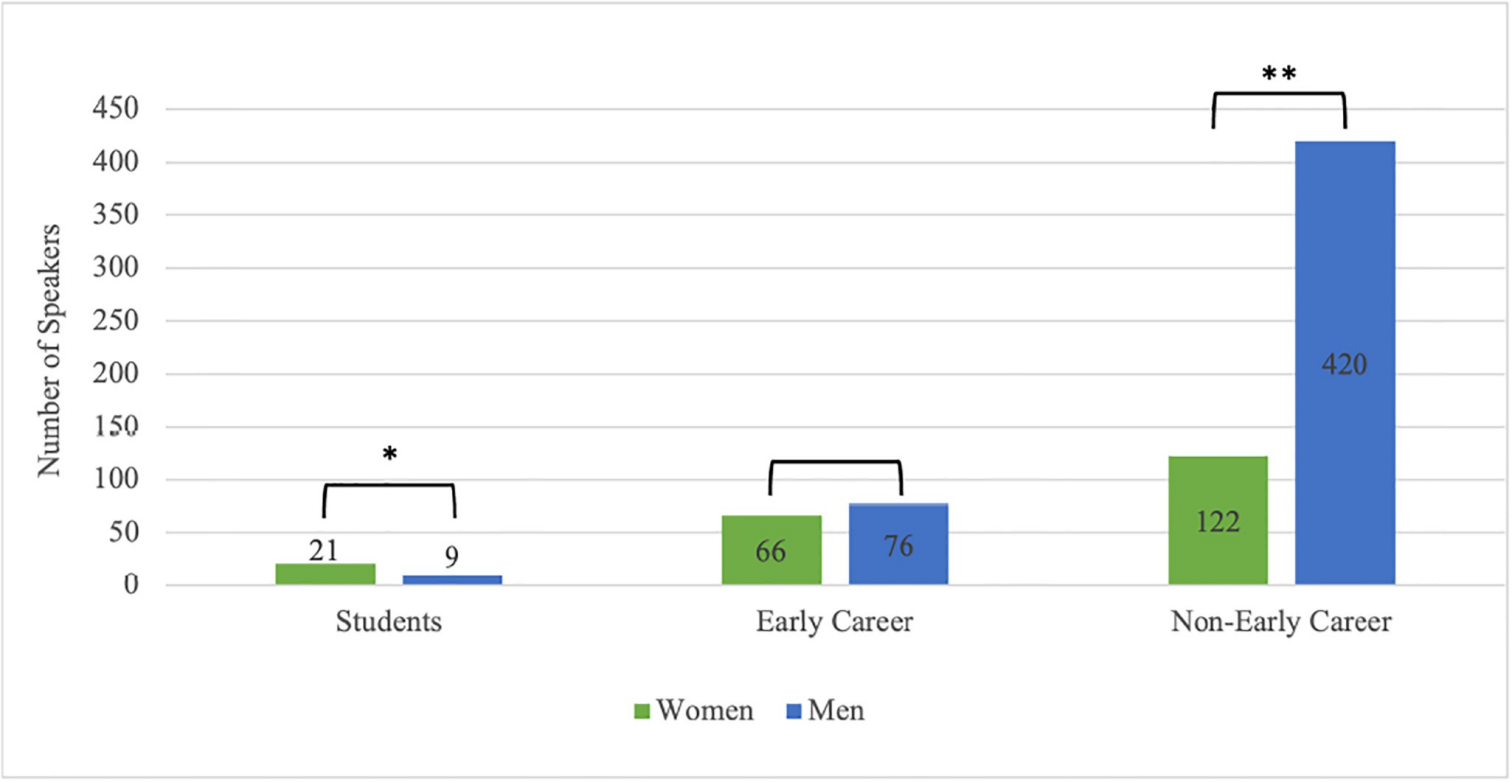
Gender representation by career stage. Note: * = *p* < .05, ** *p* < .001.

Among student speakers there were significantly more women than men $X^{2}$ (1, *N* = 30) = 4.80, *p* < .029 (Fig 4). No significant gender difference was found for early career researchers, $X^{2}$ (1, *N* = 142) = 0.70, *p* = .40. There were significantly more men than women among more senior speakers within prestigious speaking roles at gambling studies conferences, $X^{2}$ (1, *N* = 542) = 163.85, *p* < .001.

## Discussion

This is the first study to examine the distribution of prestigious speaking roles by gender at gambling studies conferences. Results demonstrate a pattern of gender disparity, with women being significantly underrepresented across speaker continent, speaker role, conference location, time, and across the majority of conferences included. Women on average held prestigious speaking roles less frequently than men and the top ten most frequent speakers yielded a ratio of nine men per woman. This historic and pervasive underrepresentation of women speakers aligns with findings from similar studies outside the gambling studies field [5,26,27]. In light of these findings, it is imperative that the gambling research field acknowledges this pressing issue and begins to address the issue of gender disparity.

Conference visibility is associated with women’s ability to succeed within academia [1,3,4,28,29]. For example, speaking at conferences has been observed to foster not only professional advancement for women by showcasing their leadership within the field, but also creates role models for women who are earlier in their career [28]. Conferences are also a known place for the germination of mentor-mentee relationships [29].

The causes of gender disparities are complex and multifaceted. Women are not only less likely to submit abstracts for oral presentations, but they are also less likely to be accepted [3,12]. Women also bear a greater share of the social and familial responsibilities that could prevent them from attending, and especially travelling to, conferences [26,27,30]. This might explain, in part, why women are less likely to accept prestigious speaking invitations [3,11]. Indeed, research has found that female early career faculty report that childcare responsibilities hinder their ability to present at conferences [4].

Our study also provides evidence of the existence of a “leaky pipeline” within the gambling studies field. The “leaky pipeline” refers to the disproportionate attrition of women at each step up the academic ladder [3,27,31]. Specifically, our study demonstrates that there are more women than men among student speakers, no significant gender disparity among early career researchers, yet a significantly lower proportion of women than men among speakers who hold more senior academic positions. In line with these findings, a recent study demonstrated that gender parity is observed in the attribution of annual graduate-level scholarships by a gambling-specific scholarship provider [24], while another presented evidence of men being overrepresented within the top 10 most-cited active researchers in the gambling studies field [20]. The leaky pipeline points to the pervasiveness of gender inequity throughout academic culture, scientific structure, and society more broadly [30]. This evidence of a leaky pipeline within the gambling studies field is concerning given that poor gender representation across the academic spectrum impacts not only current but future generations of academics and students [27].

Moving forward, as a field, we need to find ways to reduce the gender gap and increase the number of women in prestigious speaking roles at gambling studies conferences. Research has established that, because of network effects, conferences reliably include more women when there are more women on the organizing committee [5,12,32,33]. Specifically, it has been documented that until parity is achieved, the proportion of women among invited speakers at conferences increases alongside the proportion of women on organizing committees [34]. Casadevall (2015) also reported that gender equity for oral presentations given at a scientific meeting was successfully achieved by increasing women’s representation on the organizing committee, presenting the committee with gender statistics, and explicitly aiming to avoid sessions comprised exclusively of men [35]. Thus, while it is important that women are encouraged to take part as invited speakers, it is also important that conference committees adopt evidence-based strategies for improving parity, including seeking out women members, understanding their historical gender statistics, and avoiding men-only panels.

Previous studies outside the field have recommended setting clear equity targets (e.g., gender quotas) for not only conference speakers, but also conference organizing committees as well as prioritizing, collecting, and evaluating (e.g., periodically auditing) comprehensive gender-specific data [1,2,26,30]. Organizers should also select and invite women speakers first to avoid potential cancellations that could lead to an imbalance in gender representation [30]. Specific to our findings related to career stage, it is imperative that conference organizers in the gambling studies field become more supportive of the success and advancement of women scholars, especially in early stages of their careers in order to offset the attrition of women via the leaky pipeline. Given how little is understood about the specific reasons for such pervasive gender disparity within gambling studies conferences, and its potentially far-reaching consequences, it is critical that future research be dedicated to directly engaging with women (e.g., exploring their lived experiences) to discuss issues related to gender disparity at gambling studies conferences. Exploring these issues will allow for conference organizers to apply efficient measures to reduce barriers, increase support, and facilitate increased inclusion of women.

Some limitations of the present study should be noted. First, we recognize the possibility for error in categorizing gender by inference based on pronouns used in public-facing materials. While pronouns do not always correlate directly to gender and the broad spectrum of gender identities and expressions are not adequately captured by pronoun use, we chose this method to reflect the language individuals used. As our data extraction only yielded individuals who used he/him or she/her pronouns, future research addressing the lack of diversity of gender representation beyond the gender binary in the field of gambling studies is needed. The analysis was, at times, limited by the existing lack of representation (e.g., when, for certain conference locations, the number of women presenting in prestigious speaker roles was too small to even allow for statistically viable analyses to be run). Data about speakers also only included those who accepted invitations to speak as information about the invited speakers who declined was not available. As such, further research should seek to better understand the reasons why women decline in order to make opportunities more equitable.

## Conclusions

The overall picture of gender representation at gambling studies conferences indicates an urgent need for a commitment to improve gender parity within the field. Across the board, whether we are talking about conference location, speaker continent, speaker role, time, frequency, and the vast majority of conferences, women are underrepresented in prestigious speaking roles at conferences within the gambling studies field. Our findings highlight the existence of a pervasive pattern of disparity in gender representation and represent a starting point from which we, as a field, must acknowledge and move forward from to achieve gender equity. Gambling studies should begin to explore the reasons for gender disparity while proactively setting in motion the implementation of evidence-based strategies to increase the visibility of women’s contributions, counteract existing widespread gender disparity, and cultivate diversity and inclusivity within the field.
